# Early Change in Metabolic Tumor Heterogeneity during Chemoradiotherapy and Its Prognostic Value for Patients with Locally Advanced Non-Small Cell Lung Cancer

**DOI:** 10.1371/journal.pone.0157836

**Published:** 2016-06-20

**Authors:** Xinzhe Dong, Xiaorong Sun, Lu Sun, Peter G. Maxim, Lei Xing, Yong Huang, Wenwu Li, Honglin Wan, Xianguang Zhao, Ligang Xing, Jinming Yu

**Affiliations:** 1 Department of Radiation Oncology, Shandong Cancer Hospital, Shandong University, Jinan, Shandong, China; 2 Key Laboratory of Radiation Oncology of Shandong Province, Shandong Cancer Hospital and Institute, Jinan, Shandong, China; 3 Department of Radiology, Shandong Cancer Hospital and Institute, Jinan, Shandong, China; 4 Jinan University, Jinan, Shandong, China; 5 Department of Radiation Oncology and Cancer Institute, Stanford University School of Medicine, Stanford, California, United States of America; 6 College of Physics and Electronic Science, Shandong Normal University, Jinan, Shandong, China; Wayne State University, UNITED STATES

## Abstract

**Introduction:**

To observe the early change of metabolic tumor heterogeneity during chemoradiotherapy and to determine its prognostic value for patients with locally advanced non-small cell lung cancer (NSCLC).

**Methods:**

From January 2007 to March 2010, 58 patients with NSCLC were included who were received 18F-fluorodeoxyglucose (^18^F-FDG) PET/CT before and following 40 Gy radiotherapy with the concurrent cisplatin-based chemotherapy (CCRT). Primary tumor FDG uptake heterogeneity was determined using global and local scale textural features extracted from standardized uptake value (SUV) histogram analysis (coefficient of variation [COV], skewness, kurtosis, area under the curve of the cumulative SUV histogram [AUC-CSH]) and normalized gray-level co-occurrence matrix (contrast, dissimilarity, entropy, homogeneity). SUV_max_ and metabolic tumor volume (MTV) were also evaluated. Correlations were analyzed between parameters on baseline or during treatments with tumor response, progression-free survival (PFS), and overall survival (OS).

**Results:**

Compared with non-responders, responders showed significantly greater pre-treatment COV, contrast and MTV (AUC = 0.781, 0.804, 0.686, respectively). Receiver-operating-characteristic curve analysis showed that early change of tumor textural analysis serves as a response predictor with higher sensitivity (73.2%~92.1%) and specificity (80.0%~83.6%) than baseline parameters. Change in AUC-CSH and dissimilarity during CCRT could also predict response with optimal cut-off values (33.0% and 28.7%, respectively). The patients with greater changes in contrast and AUC-CSH had significantly higher 5-year OS (*P* = 0.008, *P* = 0.034) and PFS (*P* = 0.007, *P* = 0.039). In multivariate analysis, only change in contrast was found as the independent prognostic factor of PFS (*HR* 0.476, *P* = 0.021) and OS (*HR* 0.519, *P* = 0.015).

**Conclusions:**

The metabolic tumor heterogeneity change during CCRT characterized by global and local scale textural features may be valuable for predicting treatment response and survival for patients with locally advanced NSCLC.

## Introduction

Concurrent chemoradiotherapy (CCRT) is the standard of care in patients with locally advanced (stage III, inoperable) non-small cell lung cancer (NSCLC) [1]. However, even using escalated radiotherapy dose to 74Gy and adding cetuximab, no benefit in overall survival was obtained for these patients [2]. Patients with locally advanced NSCLC are a very heterogeneous population with varying degrees of tumor biology, comorbidity, and other characteristics. Therefore, a need arises to predict treatment response and long-term outcome at the early phase. By better stratification of patients, it could possibly result in improved tumor control and reduced side effects, and eventually avoidance of futile costs of ineffective treatments [3].

Efforts have been made to address this issue by identify prognostic signatures using functional imaging approaches such as ^18^F-fluorodeoxyglucose (FDG) positron emission tomography (PET) [4]. Quantification of tumor metabolism by means of standardized uptake value (SUV) is now widely used and a number of studies have demonstrated the prognostic value of tumor PET SUV obtained either before treatments, after treatments or by measuring early change during treatments [4–6]. However, no relationship between baseline SUV and outcome was found in other studies. It remains unclear whether SUV is an independent prognostic factor [5]. Previous research also describes metabolic tumor volume (MTV) and the total lesion glycolysis (TLG) using semiautomatic segmentation methods based on PET for prognostic parameters [7]. It has been shown that pretreatment MTV is a predictor of clinical outcomes for NSCLC patients treated with chemoradiotherapy [8]. The degree of change in MTV and TLG was reported to be predictive for response and long-term survival after CCRT [6,9].

Quantification of intratumoral ^18^F-FDG uptake heterogeneity has recently generated interest to predict the treatment response [10]. Kang et al reported that intratumoral metabolic heterogeneity in FDG PET could predict disease progression after CCRT in inoperable stage III NSCLC, which defined by the area under the curve of the cumulative SUV-volume histograms (AUC-CSH) [11]. Pretreatment PET features including histogram, shape and volume and co-occurrence matric features were associated with overall survival when adjusting for conventional prognostic factor in NSCLC [12,13,14]. However, to our knowledge, there was no report of change in heterogeneity features at ^18^F-FDG PET in NSCLC receiving CCRT. The purpose of our study was to observe the early change of metabolic tumor heterogeneity during CCRT and to determine its prognostic value for patients with locally advanced NSCLC.

## Materials and Methods

### Patients

This study was approved by the institutional review board at Shandong Cancer Hospital. Informed consent was waived due to the retrospective design of the study. All patient record and information was anonymized and de-identified prior to analysis. From 1^st^ November 2015, clinical data was collected. Authors only have access to collect anonymous patient information. Patients were recruited with eligibility criteria as: (1) NSCLC confirmed by histological or cytological diagnosis, (2) stage III (TNM sixth edition, UICC), inoperable or refuse operation, (3) ECOG performance status 0–1, (4) adequate normal organ function. Patients were excluded if received surgery, chemotherapy, or radiotherapy for cancer previously.

### Staging and Treatments

Routine staging procedures consisted of contrast-enhanced CT of the chest and abdomen, magnetic resonance imaging of the brain and whole-body ^18^F-FDG PET/CT scanning. Radiation was delivered using intensity-modulated radiotherapy (IMRT) or 3-dimensional conformal radiotherapy (3D-CRT) techniques. Late course accelerated hyper fractionated radiotherapy was performed as 2Gy/fractionation/day to 40Gy and 1.4 Gy twice daily to a total dose of 62.4–68.0Gy [6]. Concomitant chemotherapy consisted of 2 cycles of cisplatin-based regimen containing paclitaxel, pemetrexed, vinorelbine, or etoposide. 2–4 cycles of consolidation chemotherapy were given in 42 patients.

### 18F-FDG PET/CT Scan

Two PET/CT scans were performed for each patient. One was baseline for the initial staging and another during treatments (40Gy radiotherapy). The time between two PET/CT scans was 28±3 days. The blood glucose level was <1.4g/L before scans for all patients. The FDG PET/CT images were obtained using a GE Discovery LS system 60 minutes (range 55–70 min) after injection of 18F-FDG (4.4 MBq/kg) with a rigid protocol [15]. CT data were acquired first (120 kV and 90mA, no contrast enhancement). PET images were subsequently reconstructed with the built-in GE Advance software, using the ordered subset expectation maximization (OSEM) algorithm with 2 iterations and 28 subsets, and a 5.0 mm full-width at half-maximum (FWHM) Gaussian post-filtering. The PET (128 × 128, pixels of 3.91 × 3.91mm, 4.25-mm slice thickness) and the CT images (512 × 512, pixels of 0.98 × 0.98mm, 5.0mm slice thickness) were systematically co-registered using the GE software.

### PET Imaging Analysis

Our previous study demonstrated that the tumor volume seen on an ^18^F-FDG PET image with a cut-off value of 3.0 was the closest to the pathologic gross tumor volume [15]. On the basis of this result, the regions equal to or greater than SUV 3.0 were selected to automatically delineate the region of interest (ROI). Two clinical oncologists with the help of a specialist radiologist adjusted the regions of interest manually by visually inspecting the primary tumor borders to avoid overlapping on adjacent ^18^F-FDG-avid structures or lesions. Nodal disease was not included in the analysis. Both SUV and tumor heterogeneity parameters were extracted from the ROI. The SUV_max_ in each ROI was determined using the whole-body attenuation corrected image. The MTV was automatically generated from the ROI in cubic centimeters (cm^3^) using the Xeleris workstation.

For assessment of tumor metabolic heterogeneity, global and local scale textural features were extracted from SUV histogram analysis and normalized gray-level co-occurrence matrix (NGLCM), respectively. The selected parameters have been widely used in PET and shown robust to depict intra-tumor heterogeneity in previous studies [10,11,16,17]. All image processing process such as ROI segmentation, denoising and texture feature extraction was performed using an in-house MATLAB code (Mathworks Inc, Natick, USA). The SUV histogram analysis was used to calculate coefficient of variation of SUVs (COV), skewness, kurtosis and area under the curve of the cumulative SUV-volume histogram (AUC-CSH) [11,18]. Four parameters, including contrast, dissimilarity, entropy and homogeneity, were calculated from the NGLCM contained three-dimensional (13 different angular directions) gray-level information, as previously described [19,20]. The definitions of NGLCM are given in S1 Table. All subsequent reported results were obtained using 64 discrete values in the resampling normalization process, which were considered sufficient given the range of SUVs encountered. The parameters in baseline scan was labelled as P1, and those in the second scan as P2. Change in percentage (ΔP %) was calculated by [(P2-P1)/P1]x100%.

### Treatment Response and Follow-up

Tumor response was assessed according to the Response Evaluation Criteria in Solid Tumors (RECIST) 1.1 [21], at 12 weeks after treatments using diagnostic contrast-enhanced CT. Complete response (CR), partial response (PR), stable disease (SD) or progress disease (PD) was recorded. The patients were followed up every 3 months at the first two years and every 6 months thereafter. Overall survival (OS) was calculated from the first day of treatment to the data of death or the last follow-up. Progression free survival (PFS) was calculated from the first day of treatment to the date of local or distal failure.

### Statistical Analysis

The statistics analysis was performed using SPSS for Mac (version 22, IBM). Data are presented as the mean ± standard deviation (SD). Difference between P1 and P2 was defined using the Wilcoxon signed-rank test or paired t test after confirming whether the parameters were normally distributed or not by the Shapiro-Wilks test. Receiver-operating characteristic (ROC) analysis was performed to estimate the optimal cut-off value for the parameters in predicting treatment response. Specificity and sensitivity were derived from areas under the ROC curves (AUC-ROC). To evaluate the prognostic value of the parameters, 5-y OS and PFS were chosen as main end-points. The survival curves were generated using the Kaplan–Meier method. The difference in survival rates among groups was compared using the log-rank test. Multivariate analysis was carried out to identify the independent prognostic factors using Cox proportional hazards regression model. All statistical tests were conducted at a two-sided level of significance as *P*<0.05.

## Results

### Patient Characteristics

From January 2007 to March 2010, fifty-eight patients (38 men and 20 women) were included with median age of 58 years. Patient demographic and clinical characteristics were listed in Table 1.

**Table 1:** Patient Clinical Characteristics and Univariate Analysis of Survival.

Patient characteristic	No. (%)	PFS		OS	
		HR (95%CI)	*P*	HR (95%CI)	*P*
**Age** (≥58)	30 (51.7%)	2.371 (1.482–4.262)	0.047	3.127(1.192–5.269)	0.032
**Gender** (Male)	38 (65.5%)	1.357 (1.526–3.682)	0.045	1.751 (0.589–2.435)	0.067
**AJCC Stage** (IIIA)	24 (41.3%)	1.352 (0.392–2.623)	0.093	1.528 (0.263–1.813)	0.298
**T Stage** (1 or 2)	25 (43.1%)	0.509 (0.241–1.872)	0.389	1.625 (0.282–2.173)	0.267
**N Stage** (0, 1 or 2)	36 (62.1%)	0.929 (0.316–1.708)	0.684	0.872 (0.355–1.806)	0.256
**Location** (Left)	18 (31.1%)	1.485 (0.771–2.638)	0.962	1.756 (0.718–1.958)	0.637
**Smoking**	47 (81.0%)	2.467 (0.977–4.392)	0.057	2.653 (1.242–5.925)	0.043
**Histology**					
Adenocarcinoma	25 (43.1%)	1.391 (1.034–2.554)	0.032	0.079 (0.005–1.154)	0.072
Squamous cell carcinoma	30 (51.7%)	1.063 (0.523–2.151)	0.053	1.356 (0.518–1.578)	0.064
Other	3 (5.2%)	0.621 (0.415–6.543)	0.305	0.684 (0.111–4.204)	0.681
**Radiotherapy techniques** (IMRT)	20 (34.5%)	0.359 (0.196–2.570)	0.278	0.773 (0.415–1.462)	0.674
**Radiotherapy dose** (≤66Gy)	43 (74.1%)	1.723 (0.291–3.130)	0.073	1.432 (0.351–2.149)	0.086
**Chemotherapy regimen**					
Cisplatin/etoposide	10 (17.2%)	1.232 (0.241–2.538)	0.756	0.727 (0.481–1.219)	0.837
Cisplatin/paclitaxel	25 (43.1%)	0.241 (0.027–2.161)	0.204	0.874 (0.433–1.765)	0.707
Cisplatin/pemetrexed	16 (27.6%)	1.307 (0.214–7.986)	0.772	2.007 (0.809–4.976)	0.133
Cisplatin/vinorelbine	7 (12.1%)	0.892 (0.229–4.395)	0.992	0.998 (0.737–3.366)	0.241

### FDG Uptake Change During CCRT

All metabolic parameters’ change at baseline and intra-treatment PET images is shown in Table 2. SUV_max_ was 17.6±10.9 at baseline and decreased 43.6%± 22.5% (4% to 72.7%) during treatment. The primary tumor MTV decreased 59.7%±21.3% (6% to 96.8%) in the middle of CCRT. The textural parameters changed in different directions and degrees. Entropy, skewness and homogeneity were normally distributed, while the other parameters were not, including SUV_max_, MTV, COV, kurtosis, contrast and AUC-CSH. For the entire group of patients, significant differences were found between baseline and intra-treatment for contrast, AUC-CSH, dissimilarity, SUV_max_, MTV and COV. During CCRT, the biggest increase (79.0%±54.6%) was found in contrast, on the other hand, COV had the biggest decline (-72.7%±4.0%).

**Table 2:** Metabolic parameters at baseline and intra-treatment PET images.

Parameters	Baseline	Intra-treatment	Change (%)	*P* Value
**SUV**_**max**_	17.6±10.9	9.6±4.3	-43.6± 22.5	0.027
**MTV**	80.4±61.8 cm^3^	31.8±20.0 cm^3^	-59.7±21.3	0.010
**Contrast**	80.8±33.5	158.9±21.8	79.0±54.6	0.001
**AUC-CSH**	0.423±0.162	0.639±0.236	45.0±31.3	0.029
**Dissimilarity**	6.1±1.6	7.8±2.9	28.2±24.8	0.042
**Entropy**	6.4±0.6	5.9±1.3	-4.8±3.9	0.682
**Kurtosis**	3.6±2.5	3.4±3.1	-5.2±2.8	0.245
**Skewness**	0.8±0.4	0.7±0.5	-10.3±35.8	0.587
**Homogeneity**	0.23±0.05	0.19±0.07	-12.3±15.9	0.483
**COV**	11.4±6.6	5.8±3.2	-72.7±4.0	0.000

### Treatment Response Analysis

**Fig 1:**
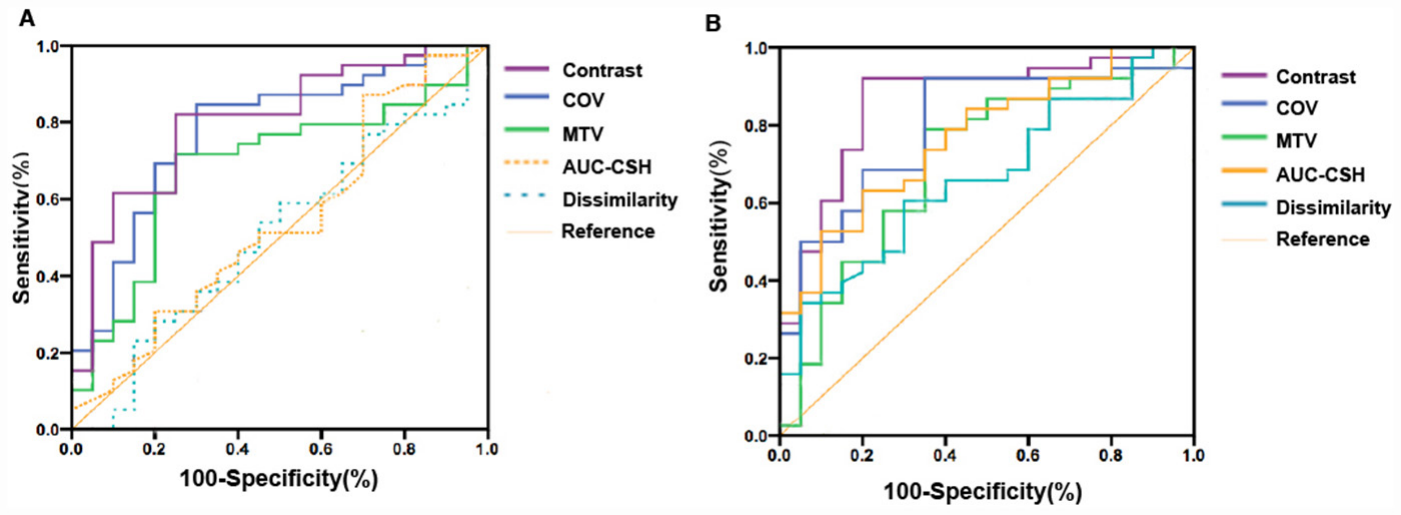
ROC curves for identifying responders vs. non-responders. ROC curves for identifying responders vs. non-responders with baseline (A) and intra-treatment change (B) of metabolic tumor heterogeneity parameters.

Thirty-eight patients (9 CR and 29 PR) were classified as responders and other 20 patients (16 SD and 4 PD) were defined as non-responders. The overall response rate was 66.2%. The capability of baseline parameters to predict tumor response was shown by the ROC in Fig 1A. The highest AUC values of ROC were found for contrast, COV, and MTV, which had the statistically significant predictive capability. Contrast and COV predicted treatment response (AUC = 0.804 and 0.781, respectively) more accurately than that MTV did (AUC = 0.686).

**Fig 2:**
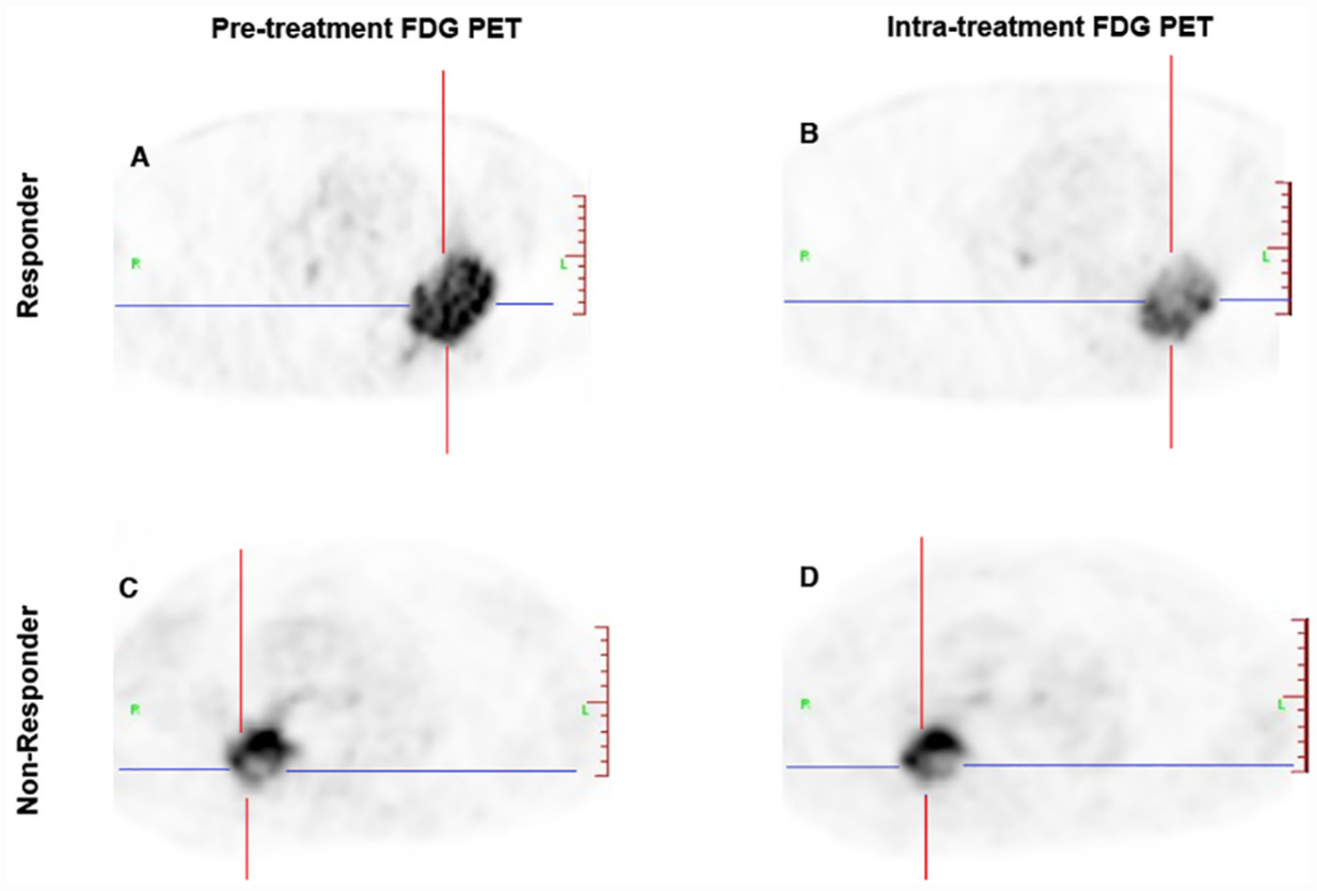
Typical examples of FDG uptake heterogeneity. Typical examples of FDG uptake heterogeneity in patients with responding (A, B) and non-responding tumors (C, D).

**Fig 3:**
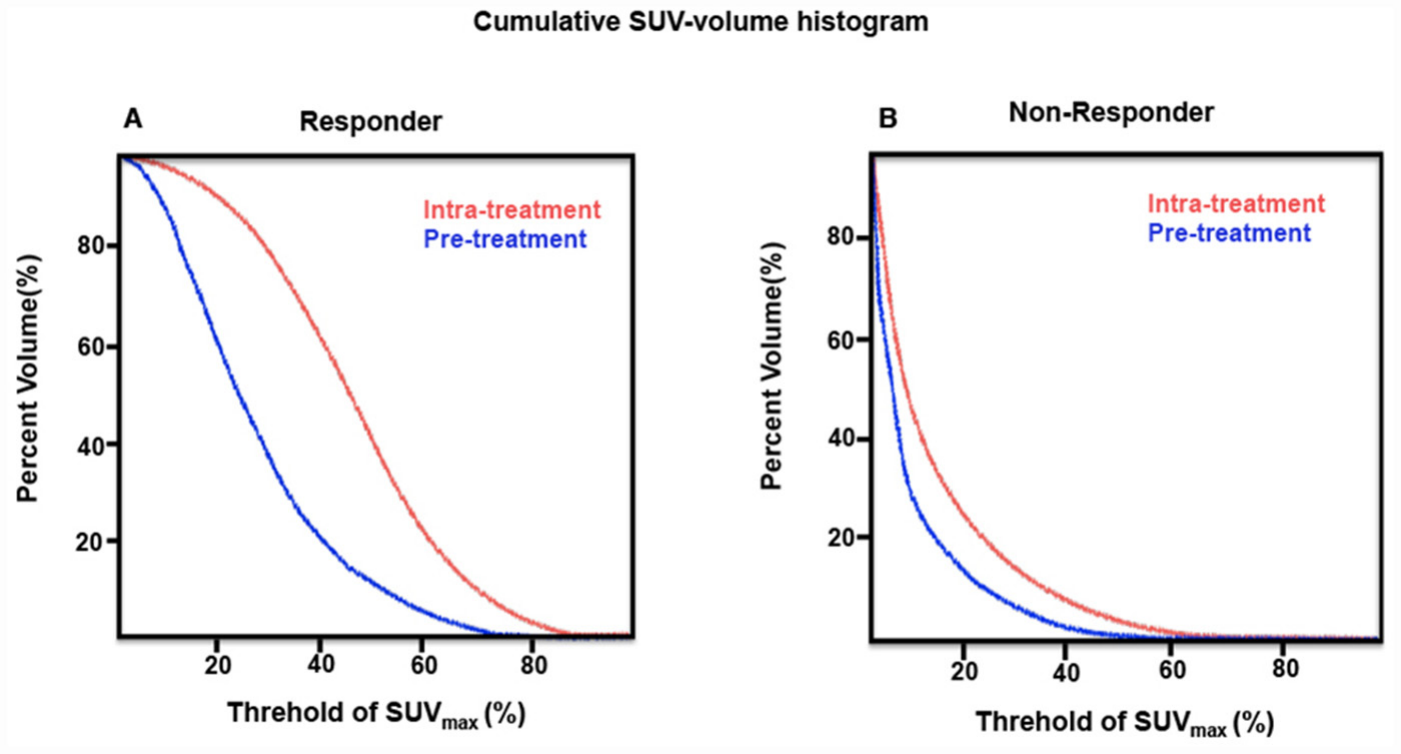
Cumulative SUV-volume histogram changes of patients in Fig 2. Compared to non-responder (B), change of AUC-CSH in the responder (A) is more obvious.

Comparing the performance of baseline PET parameters, change of tumor textural parameters during treatment could stratify non-responder and responder with higher AUC than baseline parameters, as shown in Fig 1B. The ROC curve analysis results were summarized in Table 3 as comparison of different parameters in terms of sensitivity and specificity. Δcontrast%, with an AUC of 0.862, allowed the identification of responders with a maximum sensitivity of 92.3% and specificity of 83.6%, when the threshold is set at 70.3%. Increasingly, ΔCOV% with a threshold of -58.6% also differentiated responders and non-responders with a higher sensitivity (92.1% vs 61.5%) and specificity (81.1% vs 76.2%) than the baseline values. Baseline AUC-CSH and dissimilarity were not significant predictive factors, but with optimal cut-off values (33.0% and 28.7%, respectively), ΔAUC-CSH% and Δdissimilarity% showed statistically significant predictive capability. Neither SUV_max_ nor other first- and second-order textural features extracted from the intensity histogram and NGLCM could significantly predict treatments response. Figs 2 and 3 show typical examples of metabolic heterogeneity change in PET image and cumulative SUV-volume histogram for patients with responding and non-responding tumors.

**Table 3:** The specificity, sensitivity, and AUC-ROC in predicting tumor response.

Parameters	Cut-off values	Sensitivity (%)	Specificity (%)	AUC-ROC (%)
**Baseline parameters**				
**MTV**	42.5cm^3^	71.8	74.9	0.686
**Contrast**	63.5	82.1	75.0	0.804
**COV**	6.0	61.5	76.2	0.781
**Parameters change**				
**ΔMTV%**	-57.2%	73.2	80.0	0.768
**ΔContrast%**	70.3%	92.3	83.6	0.862
**ΔCOV%**	-58.6%	92.1	81.1	0.799
**ΔAUC-CSH%**	33.0%	78.9	65.6	0.708
**ΔDissimilarity%**	28.7%	60.5	70.8	0.665

### Long-term Survival Analysis

**Fig 4:**
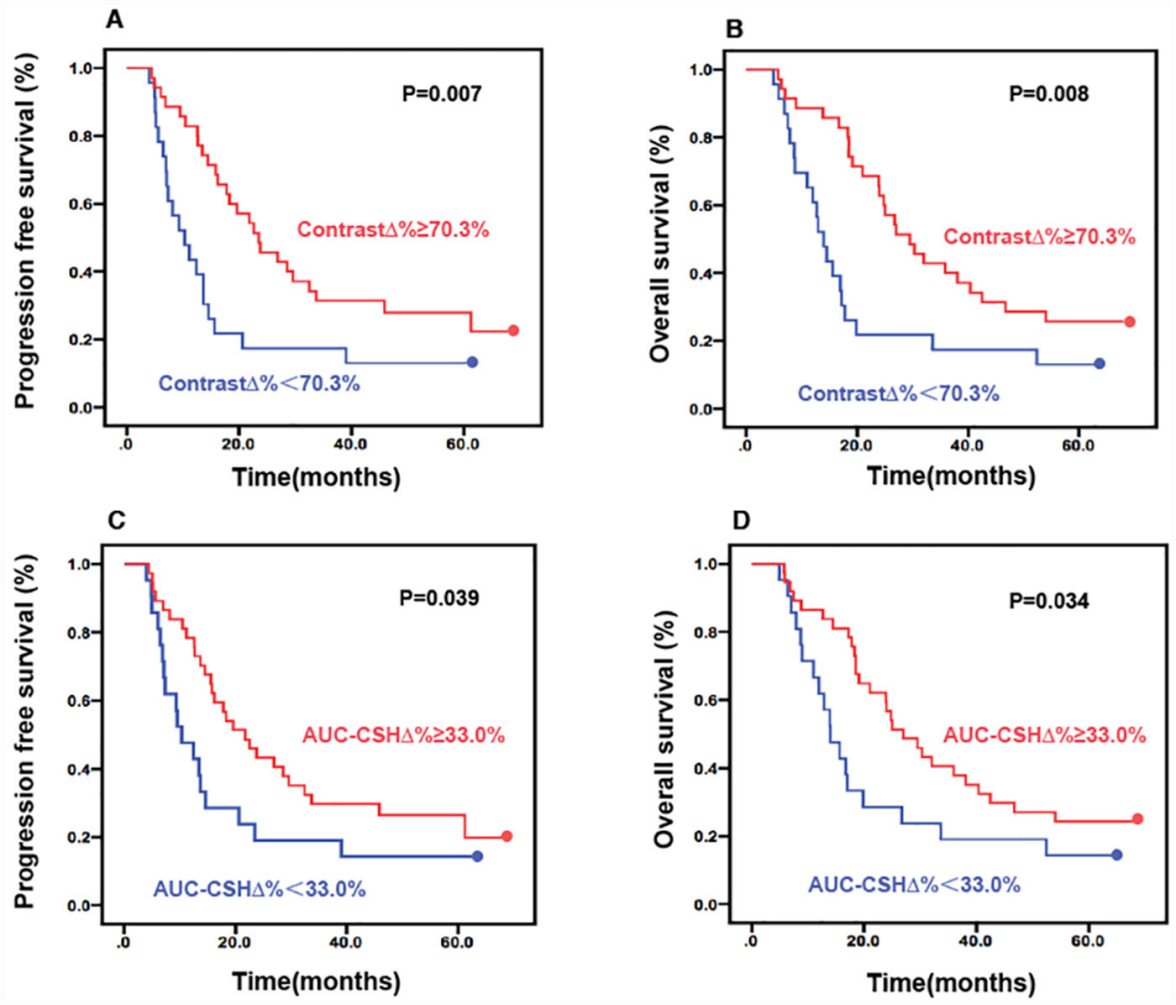
Kaplan–Meier plots for probability of PFS and OS. Kaplan–Meier plots for probability of progression-free survival (A: Δcontrast%, C: ΔAUC-CSH%) and overall survival (B: Δcontrast%, D: ΔAUC-CSH%). Time of censoring is marked by a dot.

At a median follow-up of 60 months (4.9–68 months), median PFS was 21±15.6 months with a 5-year PFS of 16%. Kaplan–Meier analysis showed that in baseline PET parameter, only contrast and COV were statistically significant prognostic factors for PFS. In addition, Δcontrast%>70.3% was associated with improved PFS with statistical significance (median PFS: 29.6 months vs.17.9 months not reached, *P* = 0.007) as shown in Fig 4A. PFS was lower in patients with lower ΔAUC-CSH% (median PFS 27.9 months vs.18.8 months not reached, *P* = 0.039), as shown in Fig 4C.

The median OS was 26±16.5 months with a 5-year OS of 21%. In the dead, 1 patient died from hepatocirrhosis related upper gastrointestinal massive hemorrhage after disease progression. One patient died in home, the precise cause of death is not specified. All other patients died from NSCLC. In the univariate analysis, both baseline contrast and Δcontrast% were significantly associated with OS. OS was shorter in patients with low Δcontrast% (median OS: 21.2 months vs. 35.1months, *P* = 0.008), as Fig 4B shown. Although the trend for baseline AUC-CSH did not significant correlate with OS statistically (*P* = 0.062), ΔAUC-CSH% was a significant prognostic factor of OS (*P* = 0.034), as shown in Fig 4D. Neither baseline nor change of other parameters could predict PFS or OS in the analysis.

Age, gender, histology, and smoke status, despite showing little prognostic potential in the univariate analyses (Table 1), were included in the multivariate model to assess any potential interactions or confounding factors. Multivariate Cox regression analysis was then used to identify the independent predictors of PFS and OS after adjustment for potential confounders. Because of the high degree of collinearity among the various PET textural features, each of them was entered separately in the multivariate Cox regression model. It was found thatΔcontrast% was the only textural feature with significant independent prognostic value for OS and PFS. Higher Δcontrast % was associated with decreased risk of tumor progression and death. The regression model analysis showed that the Odds Ratios of Δcontrast% for PFS and OS were 0.476 (95%CI 0.253–0.896, *P* = 0.021) and 0.519 (95%CI 0.267–0.997, *P* = 0.015), respectively. Univariate and multivariate survival analyses of metabolic features is shown is Table 4.

**Table 4:** Univariate and multivariate survival analyses of metabolic features.

	PFS		OS	
	HR (95%CI)	*P* Value	HR (95%CI)	*P* Value
**Univariate analysis**				
**SUV**_**max**_	2.612 (0.523–6.819)	0.118	3.484 (0.219–7.521)	0.122
**MTV**	4.587 (0.418–7.167)	0.077	5.523 (0.371–6.548)	0.165
**Contrast**	0.692 (0.146–0.924)	0.023	0.463 (0.273–0.632)	0.021
**AUC-CSH**	0.499 (0.238–1.561)	0.057	0.750 (0.339–0.805)	0.062
**Dissimilarity**	1.245 (0.792–2.129)	0.108	1.205 (0.463–1.675)	0.858
**Entropy**	2.043 (0.587–2.134)	0.154	1.114 (0.167–1.394)	0.635
**Kurtosis**	2.447 (0.484–5.359)	0.182	5.939 (0.851–7.493)	0.985
**Skewness**	1.273 (0.491–3.303)	0.097	1.136 (0.751–2.349)	0.760
**Homogeneity**	0.594 (0.293–2.270)	0.088	1.466 (0.282–2.461)	0.200
**COV**	0.432 (0.162–0.788)	0.036	0.833 (0.238–1.210)	0.075
**ΔSUV**_**max**_**%**	3.245 (0.592–5.129)	0.108	2.050 (0.632–6.755)	0.858
**ΔMTV%**	4.343 (0.587–8.134)	0.154	5.145 (0.667–7.924)	0.635
**ΔContrast%**	0.476 (0.277–0.693)	0.007	0.623 (0.242–0.995)	0.008
**ΔAUC-CSH%**	0.582 (0.149–0.758)	0.039	0.402 (0.192–0.824)	0.034
**ΔDissimilarity%**	0.952 (0.516–1.552)	0.746	0.612 (0.354–1.510)	0.098
**ΔEntropy%**	1.235 (0.721–2.138)	0.438	1.356 (0.518–1.578)	0.876
**ΔKurtosis%**	0.426 (0.322–4.644)	0.080	0.773 (0.431–1.330)	0.284
**ΔSkewness%**	1.063 (0.523–2.151)	0.879	0.724 (0.221–1.465)	0.420
**ΔHomogeneity%**	1.243 (0.578–2.646)	0.582	1.272 (0.871–2.426)	0.427
**ΔCOV%**	1.123 (0.651–2.549)	0.760	0.997 (0.651–1.293)	0.985
**Multivariate analysis**				
**Contrast**	0.723 (0.291–3.130)	0.097	0.432 (0.351–2.149)	0.760
**COV**	0.359 (0.196–2.570)	0.086	0.946 (0.522–2.061)	0.213
**ΔContrast%**	0.476 (0.253–0.896)	0.021	0.519 (0.267–0.997)	0.015
**ΔAUC-CSH%**	1.062 (0.532–2.115)	0.879	0.773 (0.415–1.462)	0.420

## Discussion

^18^F-FDG PET has been increasingly used to assess treatment response and predict patient outcome [22]. ^18^F-FDG uptake has been associated not only with increased metabolism but also other pathophysiologic factors such as perfusion, cell proliferation and hypoxia, all of which may cause for tumor heterogeneity. Therefore, the hypothesis can be made that characterizing tumor FDG distribution, through its relationship to underlying tumor biologic characterizing, may be useful in predicting treatment response [10]. In present study, we found that, in addition to the baseline parameters, temporal change of FDG uptake heterogeneity characterized by global and local textural features provided more reliable information to predict treatment response and long-term survival. Δcontrast% was not only the parameter differentiating responders and non-responders, but also the only independent prognostic factor for OS and PFS.

The underlying mechanisms, which might explain why tumor FDG uptake heterogeneity, either at baseline or change during treatments, correlated with treatments and survival, are not well established. FDG uptake is related to the expression of GLUT and hexokinase, cell proliferation, vascularization and hypoxia [23]. All these physiologic processes correlated with response to treatments [24]. One of our important finding was that higher Δcontrast % value was associated with decreased risk of progression and death. Contrast is the difference in gray scale that makes an image distinguishable. In NGLCM, contrast increase means the intensity difference between two neighboring pixels (i and j) increased (S1 Fig).

In tumor, we assume a pixel correspond to a cluster of tumor cell. High intensity in PET image pixels corresponds to high metabolic activity of tumor cells. As we know, tumors show regression of metabolic activity of during CCRT. Thus, intensity of pixel i and j decreased during CCRT. If the intensity of pixel i is higher than j in baseline PET, j must decrease much more than the neighboring pixel i did during treatment, and then the intensity difference between pixel i and j increased as contrast raise. Therefore, the hypothesis can be made that neighboring tumor cells show significant different response to CCRT due to tumor innate heterogeneity. CCRT expanded the metabolic activity gap among neighboring tumor cells. Both higher metabolic activity tumor cells and lower ones decreased during treatment, but the baseline lower metabolic tumor cells are more sensitive to CCRT than the cells with higher metabolic activity. As reflected to images, the contrast of PET increased.

Limited studies in other tumor types have investigated the predictive value of tumor metabolic heterogeneity change for assessment of therapy response. In locally advanced rectal cancer, Bundschuh et al. reported that textural parameter (COV) and its change during treatments had significant capability to assess histopathologic response and PFS, but not OS [25]. They found that higher COV indicated better histopathologic response. Besides, a decrease of COV during and after therapy indicated better histopathologic response. This is consistent with the results of our study. However, they only observed change in global scale parameters. Yang et al. found that the temporal change in the heterogeneity of intratumoral FDG distribution may provide information for understanding tumor response to chemoradiotherapy in patients with malignant cervical tumors [26]. However, only regional scale texture features were used in their study and the prognostic value of these parameters was not reported. Recently, Cook et al found that in patients with advanced NSCLC (IIIB and IV stage) treated by erlotinib, decrease in first-order entropy of FDG PET were independently associated with treatment response and OS [27]. Because of treatment-induced inflammation, the capability of SUVs in response evaluation is arguable during radiation. Measurement of heterogeneity based on ^18^F-FDG PET images and its change would provide at least a complement for response assessment and prognostic prediction.

Our results also add new evidence that textural features of ^18^F-FDG uptake within pre-treatment PET images can predict response and survival. Cook et al. used contrast in pre-therapeutic ^18^F-FDG PET to assess the tumor response to chemoradiation in 53 patients with NSCLC [28]. Compared with non-responders, RECIST responders showed higher contrast. Although the trend for contrast to predict OS did not reach statistical significance, PFS were longer in patients with high contrast (*P* = 0.015). However, none of any SUV parameters predicted RECIST responds and survival. Recently, Lovinfosse et al. also found that textural feature measured on the baseline 18F-FDG PET/CT appears to be a strong independent predictor of the outcome in patients with NSCLC treated by SBRT [14]. As shown in present study tumor FDG uptake heterogeneity indices were better parameters for predicting response and survival than the conventional ones.

Clinically, there is controversy about the use of multiple time-point imaging for treatment guidance. Using baseline PET imaging is ideal, but confront ethical challenges. It is more useful and safe to use post-treatment PET images [4], but might be too late for salvage therapy. At clinical scenario, more attention should be paid to the early changes of FDG uptake during treatment, as attempts in response-adapted treatments for lymphoma and breast cancer [29–31].

Several methodologies have been proposed to assess intratumoral FDG uptake heterogeneity and its correlation with the treatment outcome, include visual scoring [32], COV [25], AUC-CSH [11], and textural features analysis [10]. There is no consensus on the best way to define the intra-tumor FDG uptake heterogeneity. We will adapt more textural parameters in further studies. Histogram indices were highly correlated with metabolic volume, whereas some the texture indices were robust with respect to tumor segmentation [33]. Meanwhile, textural features constitute an objective heterogeneity quantification, with reduced inter-observer variability [34]. More importantly, textural analysis can also be used for CT or MR images [35]. It was demonstrated that textural parameters derived from CT images of NSCLC have the potential to serve as imaging biomarkers for tumor hypoxia and angiogenesis [36]. The nomogram from PET and CT images improved stratification amongst patients with stage II and III NSCLC, allowing identification of patients with the poorest prognosis [37]. Pretreatment CT imaging texture features could also provide prognostic information beyond that obtained from conventional prognostic factors for patients with stage III NSCLC [38]. We are conducting a study to cooperate imaging (PET and CT) parameters with conventional factors (the 7^th^ edition AJCC stage, performance status, etc) for a prognostic model for NSCLC, as reported by Vaidya et al [39] and Fried et al [12].

One major limitation of the present study was the relatively small number of patients included. Consequently, it is important to confirm our findings in larger study cohorts. Another limitation of our study is that we only analyzed the primary tumor. Including the lymph nodes could be important because of its impact on prognosis. However, considering the limited spatial resolution in PET imaging, it could be meaningless to assess FDG uptake heterogeneity on small structures such as lymph nodes [40]. Finally, the ROI for textural analysis in our study were automatically delineated with a fixed threshold and adjusted manually. For post-radiation infiltrates, we didn’t adjust the regions manually. It is possible that inter- and intra- observer variation would be reduced if more advanced segmentation technique were used, particularly for multicenter prospective studies in the future. For comparison among different research, the basic analysis procedure of texture feature should also be unified too.

## Conclusions

We demonstrated that the metabolic tumor heterogeneity changes during CCRT characterized by global and local scale textural features may provide independent information to predict treatment response and survival for patients with locally advanced NSCLC. Change in imaging contrast is not the only parameter differentiating responders from non-responders. However, it serves as the only independent prognostic factor for OS and PFS. Our results suggest that characterization of FDG PET uptake heterogeneity early during treatment holds the potential to revolutionize the predictive role of PET in personalized treatment for locally advanced NSCLC.

## Supporting Information

S1 Fig A model of change in imaging intensity and contrast after treatments. At baseline image, intensity of pixel i is higher than pixel j. For intra-treatment image, the intensity of pixel i & j decreased. But, the original lower intensity pixel j decreased much more than pixel i. Therefore, contrast of image increased.
(TIF)

S1 Table Formulas for normalized gray-level co-occurrence matrix texture parameters. From each of the primary tumor, we got one GLCM, the element of GLCM contains the number of incidences having intensity values i and j occur in two voxels separated by distance (d) in direction (a). In our implementation d was set to a single voxel size, and a was selected to cover the 13-connected neighborhood in 3D space.
(PDF)
