# Supplementary figures and images for: Early Change in Metabolic Tumor Heterogeneity during Chemoradiotherapy and Its Prognostic Value for Patients with Locally Advanced Non-Small Cell Lung Cancer
Source: PLoS One. 2016 Jun 20;11(6):e0157836. doi: 10.1371/journal.pone.0157836 (PMC4913903; doi:10.1371/journal.pone.0157836)

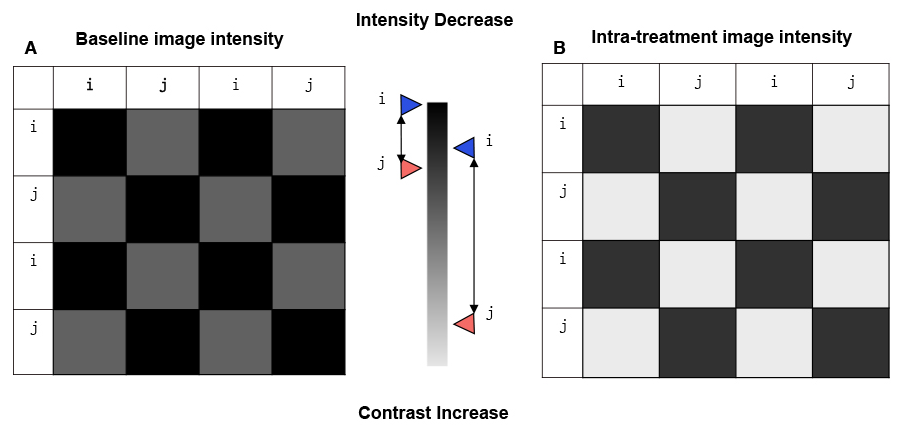

Supplement: S1 Fig — At baseline image, intensity of pixel i is higher than pixel j. For intra-treatment image, the intensity of pixel i & j decreased. But, the original lower intensity pixel j decreased much more than pixel i. Therefore, contrast of image increased. (TIF) [file pone.0157836.s001.tif]
