# Supplementary material for: Early Change in Metabolic Tumor Heterogeneity during Chemoradiotherapy and Its Prognostic Value for Patients with Locally Advanced Non-Small Cell Lung Cancer
Source: PLoS One. 2016 Jun 20;11(6):e0157836. doi: 10.1371/journal.pone.0157836 (PMC4913903; doi:10.1371/journal.pone.0157836)
Supplement: S1 Table — From each of the primary tumor, we got one GLCM, the element of GLCM contains the number of incidences having intensity values i and j occur in two voxels separated by distance (d) in direction (a). In our implementation d was set to a single voxel size, and a was selected to cover the 13-connected neighborhood in 3D space. (PDF) [file pone.0157836.s002.pdf]

Co-occurrence matrix	Formulas
**Contrast**	$\sum_i \sum_j (i-j)^2 P(i,j)$
**Dissimilarity**	$\sum_i \sum_j \lvert i-j \rvert P(i,j)$
**Entropy**	$-\sum_i \sum_j P(i,j) \log P(i,j)$
**Homogeneity**	$\sum_i \sum_j P(i,j)/(1+\lvert i-j \rvert)$
